# Modeling Olfactory Bulb Evolution through Primate Phylogeny

**DOI:** 10.1371/journal.pone.0113904

**Published:** 2014-11-26

**Authors:** Steven Heritage

**Affiliations:** Interdepartmental Doctoral Program in Anthropological Sciences, Stony Brook University, Stony Brook, New York, United States of America; Duke University, United States of America

## Abstract

Adaptive characterizations of primates have usually included a reduction in olfactory sensitivity. However, this inference of derivation and directionality assumes an ancestral state of olfaction, usually by comparison to a group of extant non-primate mammals. Thus, the accuracy of the inference depends on the assumed ancestral state. Here I present a phylogenetic model of continuous trait evolution that reconstructs olfactory bulb volumes for ancestral nodes of primates and mammal outgroups. Parent-daughter comparisons suggest that, relative to the ancestral euarchontan, the crown-primate node is plesiomorphic and that derived reduction in olfactory sensitivity is an attribute of the haplorhine lineage. The model also suggests a derived increase in olfactory sensitivity at the strepsirrhine node. This oppositional diversification of the strepsirrhine and haplorhine lineages from an intermediate and non-derived ancestor is inconsistent with a characterization of graded reduction through primate evolution.

## Introduction

In mammals, olfactory receptor neurons situated in the olfactory epithelium of the nasal cavity send axons through cribriform foramina of the ethmoid bone and enter the olfactory fossa. Here these axons synapse with mitral cells located in the main olfactory bulbs (OB) of the telencephalon. When odorant molecules bind to membrane proteins of olfactory receptor neurons, an action potential is triggered and relayed by mitral cells to the pyriform cortex and other brain areas. Olfactory sensitivity (the detection threshold of a concentration of odorant molecules) is related to the number of receptors in the olfactory epithelium and the physical size of the OB is correlated with this receptor neuron population size [1]–[3]. On this basis, interspecific studies of mammalian olfaction have typically used OB volume as a proxy for the magnitude of olfactory sensitivity. Compared to other tetrapods, olfaction is elaborated in mammals and this trait is evident in the basal-most mammaliaforms who evolved distinctly larger OBs than their cynodont predecessors [4].

**Figure 1:**
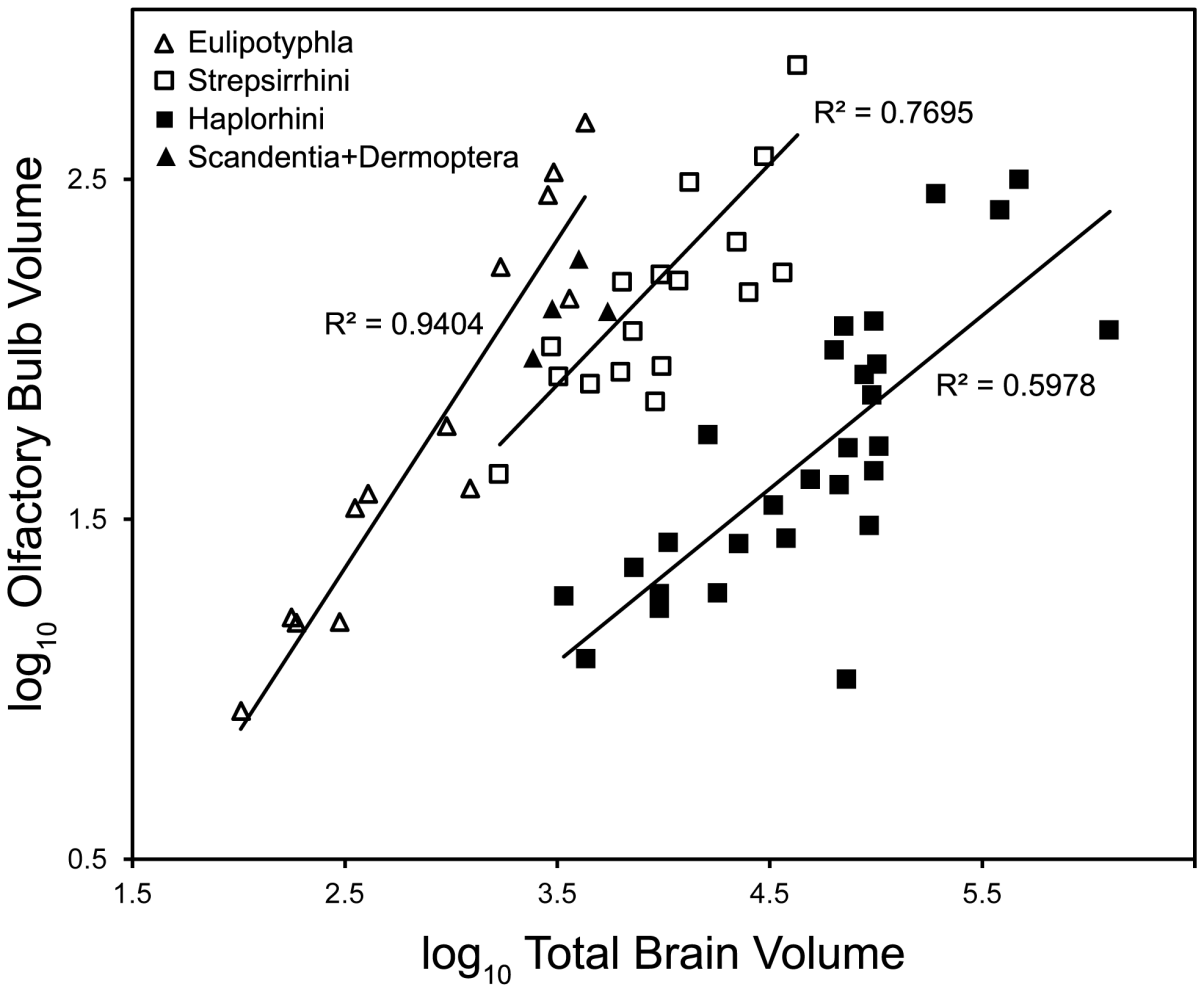
Scatter plot of log transformed OB and TB volumes. Data points are observed values. Regression lines are PGLS linear models based on extant taxa from the strepsirrhine, haplorhine and eulipotyphlan clades. Scandentia + Dermoptera do not contribute to regressions.

The primate order has been characterized as microsmatic (low olfactory sensitivity) relative to other mammals [2]. However, some authors have suggested that only the haplorhine suborder is microsmatic [5], [6]. A plot of OB volume relative to total brain (TB) volume (Fig. 1) depicts 'grade-shifts' between haplorhines, strepsirrhines and non-primate insectivorous mammals. The explanation usually posited for the distribution of these data is (a) that extant insectivorous mammals represent the ancestral state for mammals, (b) that relative to other mammals, primates evolved a reduction in olfactory sensitivity and (c) that relative to the strepsirrhine suborder, haplorhines evolved a further reduction.

In a phylogenetic context, this interpretation implies two synapomorphies: a reduction in relative OB size for primates and a further reduction for haplorhines. It also implies that the plesiomorphic state of mammalian olfactory sensitivity is known. A plotted group of terminal taxa sets up a comparison between groups rather than between a clade and its ancestral node making it difficult to gauge directionality in trait evolution. It is also difficult to detect whether sister-clades nested within a plotted group have independently evolved similar trait metrics.

Some aspects of primate biology suggest that OB evolution may have been more complex than grade-shifts. For instance, there is some evidence that ancestral anthropoids had relatively small brains and that encephalization in platyrrhines and catarrhines is convergent [7], [8]. If these sister-clades independently increased TB volumes, then their OBs (part of the telencephalon) may have independently achieved similar relative volumes. Moreover, the relative OB size of the anthropoid common ancestor may have been considerably larger if its TB volume was smaller. From the fossil record, OB and TB volumes have been estimated for a few primate taxa whose phylogenetic positioning near the base of extant clades may inform the ancestral state of olfactory sensitivity. For instance, *Parapithecus grangeri*, a stem-anthropoid from the Oligocene of Egypt has been described as having a relatively large OB [9]. If this is plesiomorphic to the anthropoid group, it may also suggest that platyrrhines and catarrhines are convergent microsmats [10]. Additionally, the relative OB size of *Rooneyia viejaensis* [11] from the late Eocene of Texas is smaller than known Eocene strepsirrhines and tarsiiforms [12] but larger than known Oligocene anthropoids [9], [13]. While the phylogenetic placement of *Rooneyia* is uncertain, if it is basal among crown-primates as recently proposed [11], major primate lineages may have evolved both increases and decreases in olfactory sensitivity rather than a few stepped reductions. The matter may be further complicated by the catarrhine loss of the vomeronasal organ (VNO) and associated accessory bulb (sometimes reported as vestiges). All other primate groups retain this sensory anatomy [10], [14]. In catarrhines, the lack of a VNO may or may not influence selection pressure on primary olfaction and the size of the main OB. In addition, nocturnality has been hypothesized as a factor in selection for keener olfactory sensitivity [15]. It is interesting that the nocturnal strepsirrhine *Daubentonia* has the largest olfactory bulb of any primate, irrespective of TB volume (Table S1 in File S1). Nearly all taxa of the haplorhine clade (except *Tarsius* and *Aotus*) are diurnal. Selective release concomitant with diurnality may be an explanation for the proportionately smaller OBs of haplorhines [16]. However, many strepsirrhines are also diurnal so this explanation is not universal. Regardless of variables that might explain how OBs have evolved differentially across the order, some principal ideas about primate olfaction are still being discussed. Some authors have questioned if olfactory sensitivity is better gauged by absolute or relative measures of OB volume (see discussion). Others have argued that some primates, assumed to be microsmats, are more sensitive to odorants than was previously thought [17].

This study analyzes the evolution of primate olfactory bulbs in a phylogenetic framework. Using a Bayesian approach, OB and TB volumes are reconstructed for ancestral nodes of major primate clades. Values at parent nodes are then compared to daughter nodes to assess the pattern of change in olfactory sensitivity through primate evolution.

## Methods

OB and TB volumes for extant taxa are from Stephan et al. [18] and Pirlot and Kamiya [19] and these OB values exclude the accessory bulb. Some names in the extant dataset were converted to reflect current species rank taxonomy [20]. Stephan et al. [18] obtained volumetric measurements of brain components following the methods used by Stephan and Pirlot [21] and Stephan et al. [22]. In this process, the bulbus olfactorius (main OB) was histologically separated from the bulbus olfactorius accessorius (accessory bulb) and volumes of these components were reported individually. Pirlot and Kamiya [19] referenced these same methods in their quantification of *Cynocephalus* brain components but reported a volume for the bulbus olfactorius only. The methodological citation suggests histological separation but an unreported volume for bulbus olfactorius accessorius may mean that both the accessory and main bulbs were quantified together. Based on available data for living euarchontans [18], a combined volume overestimates the main OB volume by 2.2% on average. The present study assumes the *Cynocephalus* OB value excludes the accessory bulb but if this is not the case the appropriate OB volume should be slightly smaller. Estimates of OB and TB volumes for the fossil crown-primates *Adapis parisiensis*, *Notharctus tenebrosus*, *Necrolemur antiquus* and *Tetonius homunculus* are from Gurche [12], *Parapithecus grangeri* from Bush et al. [9] and *Aegyptopithecus zeuxis* from Simons et al. [13]. Volumes for the stem-primates *Ignacius graybullianus* and *Microsyops annectens* are from Silcox et al. [23] and Silcox et al. [24] respectively. For fossils, OB estimates are based on endocasts or virtual endocasts of the olfactory fossa. In this method, the posterior extent of the OB is delineated by a sharp constriction termed the circular ( =  annular) fissure and the portion of the telencephalon rostral to this landmark is taken to represent both the accessory and main bulbs. No fossil taxa in this study are crown-catarrhines so the accessory bulb and VNO were presumably present. Additionally, reconstructions from fossils do not subtract the cerebrospinal fluid filled ventricle that is variably present within the OBs of mammals [1], [9]. Given that the primate accessory bulb and olfactory ventricle are small portions of the total olfactory fossa volume, an endocast of the olfactory fossa may be a reasonable estimator for the main OB [16] but these values should be interpreted as over-approximations. The final dataset used here included 19 strepsirrhines, 32 haplorhines, 2 stem-primates, 1 dermopteran, 3 scandentians, 13 eulipotyphlans and 13 afroinsectivorans. All volumes were log_10_ transformed prior to analysis (Table S1 in File S1). OB and TB data were treated individually (rather than combined into a ratio *a priori*) so that change in both absolute and relative OB size could be assessed.

Phylogenetic topology and branch lengths were based on ultrametric, time-scaled trees (soft bounded, independent rates) from Springer et al. [25] for extant primates and the DNA analyses of Meredith et al. [26] for non-primate mammals. These studies employed similar methods in constructing phylograms from molecular supermatrices composed of multiple gene segment alignments and in constructing chronograms using a relaxed molecular clock and multiple fossil calibrations. Where these studies have taxonomic overlap, topology is fully congruent and there is a close correspondence in estimated divergence dates for major clades. For instance, Springer et al. [25] and Meredith et al. [26] respectively join: Scandentia to Primates + Dermoptera at 84.61 and 83.89 Ma, Strepsirrhini to Haplorhini at 71.36 and 71.90 Ma, Lorisoidea to Lemuroidea at 54.91 and 54.03 Ma, and Tarsiiformes to Anthropoidea at 64.18 and 66.08 Ma. Given the similarity of these trees, portions of each of them were grafted together to create a phylogeny that accommodates taxonomic sampling in the OB and TB dataset. Four subtrees were prepared prior to assembling a final phylogeny.

*Crown-primates subtree*. The Springer et al. [25] chronogram was pruned to include only the 17 extant strepsirrhines and 28 extant haplorhines in the OB and TB dataset. Fossil crown-primates (2 strepsirrhines and 4 haplorhines) were then grafted to the pruned chronogram of extant taxa. For fossil crown-primates, topological placement, diversification dates and branch terminations followed Boyer and Seiffert [27] (Fig. S1 in File S1). The position of *Necrolemur* and *Tetonius* in close relation to *Tarsius* forms a Tarsiiformes group [28]. Analysis of an alternate tree which places *Necrolemur* and *Tetonius* as stem-haplorhines [29] is in Fig. S6 in File S1.

*Eulipotyphla subtree*. The Meredith et al. [26] chronogram used species exemplars (and in some cases congeneric chimeras) to estimate family and subfamily rank diversifications. To incorporate the 13 eulipotyphlan species in the OB and TB dataset, it was necessary to estimate topology and time-scaled branch lengths below the family/subfamily ranks. Following Meredith et al. [26], a constraint topology was created for Solenodontidae, Talpidae, Erinaceidae, Soricinae and Crocidurinae. Species from the OB and TB dataset were binned into their respective taxonomic groups [20] with the assumption of monophyly at these ranks. A phylogram was estimated by fitting a 13 gene (3 mitochondrial, 10 nuclear) concatenation to the constraint. The resultant topology was consistent with Colangelo et al. [30] and Dubey et al. [31]. A chronogram was then estimated by linearizing the phylogram while maintaining four Eulipotyphla family/subfamily diversification dates from Meredith et al. [26] (Fig. S2 in File S1).

*Afroinsectivora subtree*. Afroinsectivora subtree preparation followed the method described for eulipotyphlans. Macroscelididae, Chrysochloridae, Potamogalinae and Tenrecinae + Oryzorictinae were incorporated into a constraint topology that followed Meredith et al. [26]. The 13 afroinsectivoran species from the OB and TB dataset were binned into their respective taxonomic groups. An 11 gene (2 mitochondrial, 9 nuclear) concatenation was fit to the constraint. The resultant topology was consistent with Poux et al. [32]. Four Afroinsectivora family/subfamily diversification dates from Meredith et al. [26] were maintained during linearization of the phylogram (Fig. S3 in File S1).

*Scandentia subtree*. A 14 gene (2 mitochondrial, 12 nuclear) concatenation was used to estimate a phylogram for scandentians. Topology was consistent with Roberts et al. [33] and notably resolved *Tupaia glis* as more closely related to *Urogale everetti* than to *Tupaia minor*. Linearization maintained the diversification dates for *Ptilocercus* and *Tupaia* from Meredith et al. [26] and for *T. glis* and *T. minor* from Springer et al. [25] (Fig. S4 in File S1).

**Figure 2:**
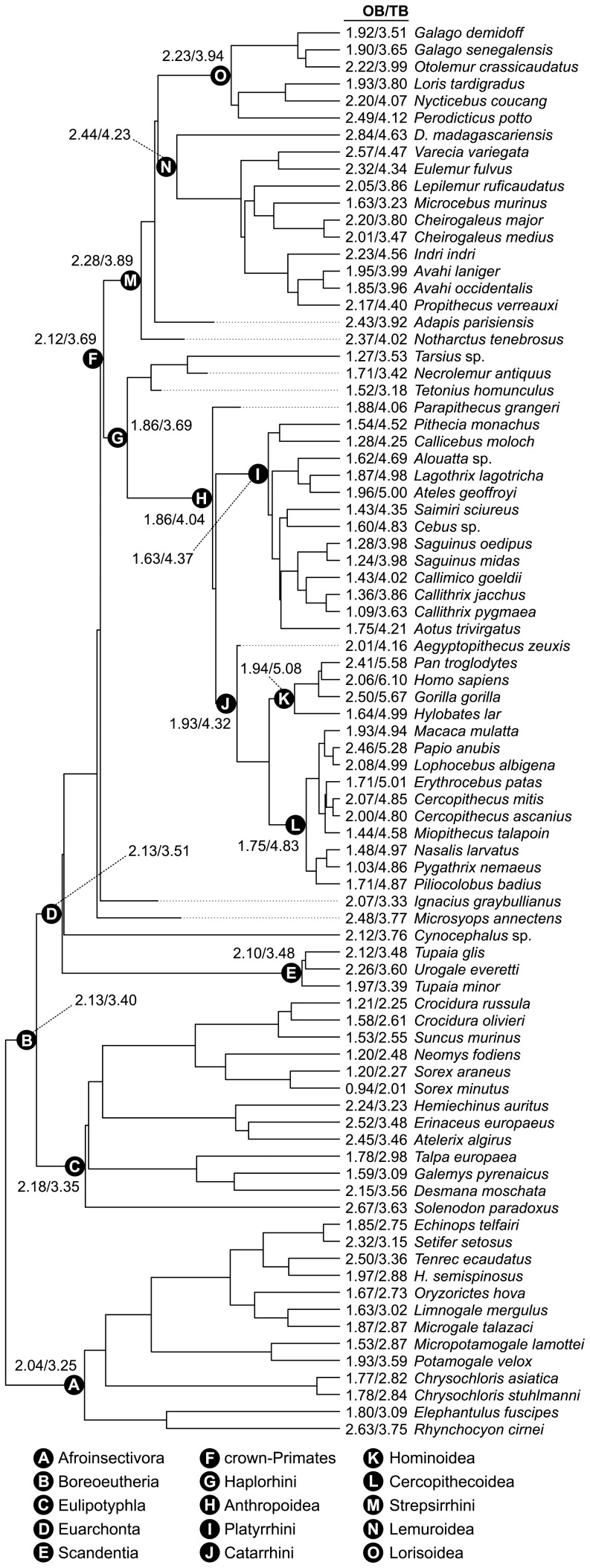
OB and TB volumes modeled through primate phylogeny. Values at terminals (OB/TB) are log transformed volumes from multiple sources (see methods). Values at ancestral nodes are mean log volumes estimated by the MCMC method. Tree is based on Springer et al. [25] for extant primates and Meredith et al. [26] for non-primate mammals and then modified to incorporate fossil taxa.

The final phylogenetic tree used in this study (Fig. 2, Fig. S5 in File S1) was assembled by grafting together the four subtrees and *Cynocephalus* (Dermoptera) following the Meredith et al. [26] chronogram. Lastly, stem-primates (*Ignacius* and *Microsyops*) were grafted to the tree following the topology of Bloch et al. [34] and with branch terminations corresponding to the age of the specimens. Similar to Boyer and Seiffert [27], ghost lineages were minimized by estimating diversification of each stem-primate branch at 1 Ma older than the base node of its sister-clade.

Accession numbers for DNA sequences retrieved from NCBI are in File S2. Sequences were aligned individually using MUSCLE v3.8 [35] and then concatenated into supermatrices (concatenation lengths: Eulipotyphla  = 11,777, Afroinsectivora  = 11,708, Scandentia  = 9,850). Phylograms for non-primate subtrees were estimated with MEGA v5.10 [36] using the maximum likelihood method and GTR + Γ+I substitution models recommended by MEGA model searches. Chronograms for non-primate subtrees were estimated with r8s v1.8 [37], [38] using the penalized likelihood method and truncated Newton algorithm.

*BayesTraits* v1.1B [39] was used to test and model continuous trait evolution. Maximum likelihood estimates of Brownian (constant variance random walk) and Directional models included all combinations of phylogenetic scaling parameters (Table S2 in File S1). Likelihood ratio tests were used to assess model fits. When comparing the likelihood (Lh) of a model with *n* parameters to a nested model with *n+1* parameters, the more complex model was considered a significantly (*P*<0.05) better fit if 2*(Lh complex model - Lh simple model) was >3.84. Brownian and Directional models that included the κ parameter significantly improved their respective non-parameter versions and also outperformed other single-parameter versions. Two and three parameter versions did not significantly outperform κ versions. Finally, the more complex Directional +κ models did not significantly outperform the Brownian +κ models. Given this analysis, the preferred models for both the OB and TB datasets were Brownian motion and included estimation of the κ scaling parameter (Table S2 in File S1).

OB and TB volumes at ancestral nodes were reconstructed individually using the Markov chain Monte Carlo (MCMC) method and the preferred models. Rate and data deviation settings were tuned to achieve parameter and ancestral estimation acceptance rates between 20–40%. For each chain (OB and TB), iterations were set to 53 million, burn-in to 3 million and sampling to every 100th step. Burn-in iterations were discarded. Tracer v1.5 [40] was used to calculate mean values and 95% lower and upper highest posterior densities (HPD).

Phylogenetic generalized least-squares (PGLS) regressions (Fig. 1, Table S3 in File S1) of log OB on log TB volumes were calculated with *BayesTraits*. For each clade (Haplorhini, Strepsirrhini, and Eulipotyphla), one version of the linear model excluded scaling parameters and three alternate versions included maximum likelihood estimates of single scaling parameters (κ, δ and 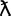). Preferred models were discerned using likelihood ratio tests (Table S3 in File S1).

## Results

For PGLS regressions, the model best fit to the haplorhine data included a zero estimate for the 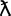 parameter. This indicates that phylogeny has no significant influence in the OB-TB relationship for the haplorhine clade (Table S3 in File S1). For trait reconstructions, the MCMC posterior distributions of κ yielded mean values of 0.36 from the OB chain and 0.27 from the TB chain (Table S4 in File S1). Values below 1.0 adjust the model by compressing long branches more than short branches indicating that the traits did not evolve gradually through the phylogeny [41].

**Figure 3:**
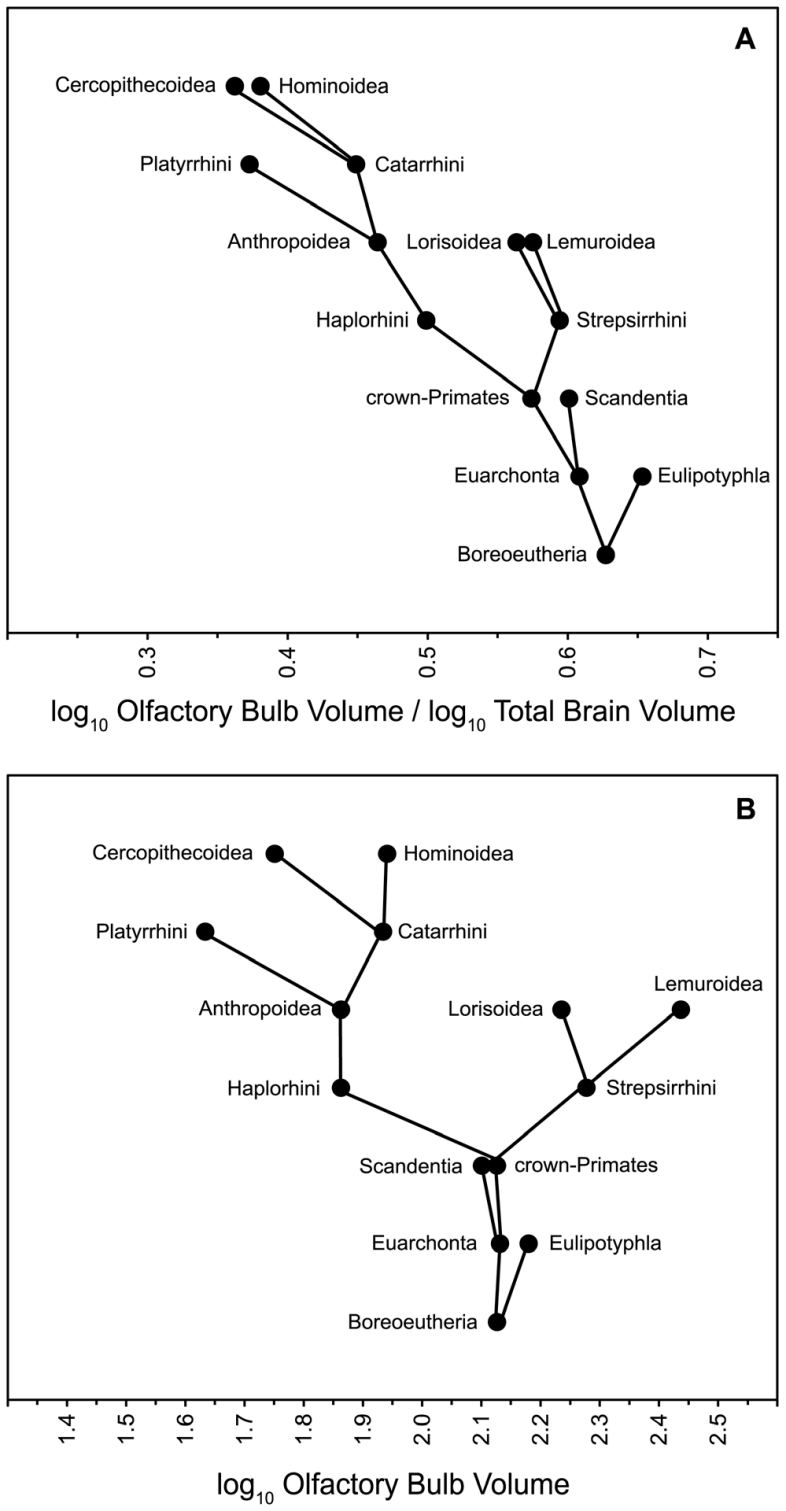
Paths of OB evolution. (A) OB change relative to TB volume. (B) Absolute OB change. Vertical axes depict clade nesting and are dimensionless.

Nodal reconstructions from the OB chain resulted in mean log volumes ranging from 1.63 (Platyrrhini) to 2.44 (Lemuroidea) and from the TB chain ranging from 3.25 (Afroinsectivora) to 5.08 (Hominoidea). Estimates of the tree root (alpha) were within the spread of the terminals as constrained by the Brownian model. Mean values for reconstructed nodes are in Fig. 2 and 95% HPDs are in Table S4 in File S1. The paths of OB evolution, from ancestral to nested nodes, are depicted in Fig. 3.

## Discussion

An ongoing challenge for comparative olfaction research is to assess the appropriateness of scaling OB volume by expressing it as a ratio to some other measurement (e.g. total brain volume or body mass). If a lineage evolves increased body size (BS), the increase in muscle tissue and skin surface area should require a greater absolute neural supply. Thus, it is expected that somatomotor and somatosensory brain elements also increase with BS [1]. Indeed, BS and TB volume are highly correlated in mammal groups and both of these traits are commonly used as scaling factors in comparative research. However, olfactory sensitivity appears to be largely related to the number of olfactory chemoreceptors in the olfactory epithelium and OB volume tracks this neuron population size [3]. Several clinical studies have shown a relationship between OB volume and olfactory sensitivity [42]–[44]. Cartmill [5] proposes that if the absolute number of olfactory receptor sites available for odorant binding is the appropriate measure of sensitivity, then BS is an irrelevant factor. Similarly, Smith and Bhatnagar [1] argue that, in an interspecific context, similarly sized OBs should signify comparable sensitivity and that applying a scaling factor such as BS or TB volume distorts the measure of olfactory function. This position is tentatively adopted here and absolute OB volume is primarily considered in conclusions about phylogenetic change and in characterizing ancestral nodes. However, OB/TB ratios are included for comparison and because there are conflicting views about applying scaling factors in interspecific studies of olfactory sensitivity. There should be little ambiguity where both absolute and relative measures support the same conclusions. Still, the number of olfactory chemoreceptors (proxied by OB volume, scaled or unscaled) may not be the only variable in a mammal's sensitivity to odorants [16].

OB volume reconstructed for the crown-primate node is comparable to the extant scandentian *T. glis*. However, *T. glis* is less encephalized than the ancestral crown-primate (Fig. 2). No primate in the dataset closely approximates OB and TB volumes at the reconstructed ordinal node. The TB model predicts independent volumetric increases for the strepsirrhine and anthropoid lineages, the platyrrhine and catarrhine lineages, and the hominoid and cercopithecoid lineages (Fig. 2). Given the results of this phylogenetic model, here I ask and answer six questions about the evolution of primate olfaction.

(1) Is the eulipotyphlan clade a suitable model for the ancestral state of olfactory sensitivity in primate ancestors? Relative to the ancestral boreoeutherian, the reconstructed eulipotyphlan node increased both absolute OB size and OB/TB ratio. Thus, olfactory sensitivity at the eulipotyphlan node may be interpreted as derived rather than plesiomorphic. In both path-plots (Fig. 3), the values for the eulipotyphlan node are right-shifted compared to its ancestor and given the sequence of cladogenesis, this position evolved independently from other plotted groups. Eulipotyphlans are distant relatives of primates and have long been subject to their own selection pressures. Consistent with the argument made by Martin [45], the reconstructed eulipotyphlan and its descendent terminal taxa are probably poor exemplars for an ancestral state of primate olfactory sensitivity.

(2) Is a synapomorphy of decreased olfactory sensitivity applicable to the crown-primate node? Relative to the ancestral euarchontan, the reconstructed crown-primate node decreased OB size slightly but increased TB size. The result is a decreased OB/TB ratio. However, the magnitude of change is mostly attributable to TB evolution (Fig. 2 and 3). Most authors agree with the assessment of Jerison [46] that encephalization has characterized the primate order since its earliest diversification. Thus, a synapomorphy of decreased olfactory sensitivity may only apply if TB size is an appropriate scaling factor.

(3) Is the strepsirrhine node plesiomorphic for olfactory sensitivity? Relative to the ancestral crown-primate, the reconstructed strepsirrhine node increased both absolute OB size and OB/TB ratio. Therefore, olfactory sensitivity at the strepsirrhine node was probably derived. Absolute OB increase between the crown-primate and strepsirrhine nodes is the largest change between any of the reconstructed parent-daughter nodes. Furthermore, OB size for the reconstructed strepsirrhine is larger than the eulipotyphlan node (Fig. 3B).

(4) Is a synapomorphy of decreased olfactory sensitivity applicable to the haplorhine or anthropoid nodes? Relative to the ancestral crown-primate, the reconstructed haplorhine decreased OB size but retained TB size (Fig. 2). Reduction in both OB size and OB/TB ratio may justify a synapomorphy of decreased olfactory sensitivity for the haplorhine node. When using the alternate phylogenetic hypothesis, TB size is not static between the crown-primate and haplorhine nodes but both analyses are consistent in predicting left-shifted OB size and OB/TB ratio (Fig. S6 in File S1). Relative to the ancestral haplorhine, the reconstructed anthropoid retained OB size but increased TB size (Fig. 2). Change in the OB/TB ratio is wholly a function of increase in the TB scaling factor. An interpretation of sensitivity reduction at the anthropoid node would depend on whether an absolute or scaled metric is used (Fig. 3). It is notable that OB sizes at the haplorhine and anthropoid nodes are smaller than most terminal strepsirrhines in the dataset. This finding compliments work by Radinsky [47] which concluded that by the emergence of crown-anthropoids like *Aegyptopithecus*, that lineage had evolved smaller olfactory bulbs and larger brains relative to most strepsirrhines.

(5) Have the platyrrhine and catarrhine lineages independently evolved decreased olfactory sensitivity? Both the OB and OB/TB path-plots predict a derived reduction from the anthropoid to the platyrrhine nodes (Fig. 3). Change through the catarrhine lineage is more complex. Relative to the ancestral anthropoid, the reconstructed catarrhine increased both OB and TB sizes (Fig. 2) but did so disproportionately resulting in a decreased OB/TB ratio (Fig. 3A). From the catarrhine node, the cercopithecoid lineage evolved a decrease in both OB size and OB/TB ratio and in each case the reconstructed cercopithecoid is left-shifted compared to the anthropoid node (Fig. 3). The reconstructed hominoid essentially retained the catarrhine OB size (Fig. 3B) but increased TB size resulting in a lower OB/TB ratio (Fig. 3A). By both absolute and relative measures, the platyrrhine and cercopithecoid nodes convergently model as reduced compared to the ancestral anthropoid. Interpretation of change from the anthropoid to catarrhine and hominoid nodes is contingent on the use of the TB scaling factor.

(6) Are fossil crown-primates consistent with a strepsirrhine/haplorhine olfaction dichotomy? Relative to the reconstructed crown-primate node, both fossil strepsirrhines in the dataset have a larger OB and OB/TB ratio. The OB sizes and OB/TB ratios for all four fossil haplorhines are smaller than at the crown-primate node. Fossil taxa are consistent with a hypothesis of oppositional derivation in olfactory sensitivity for the strepsirrhine and haplorhine lineages from an intermediately sensitive primate ancestor.

A collective characterization of the primate order as having evolved a decrease in olfactory sensitivity is inconsistent with the phylogenetic model presented here. A derived reduction better characterizes the haplorhine lineage and this interpretation is supported by both absolute and scaled metrics. Both metrics also support a derived increase for the strepsirrhine lineage as well as a crown-primate ancestor with intermediate sensitivity relative to the subordinal nodes. Relative to the ancestral euarchontan, OB size at the crown-primate node is essentially retained but this node is encephalized. Furthermore, both metrics predict a convergent decrease in the platyrrhine and cercopithecoid lineages. The treatment of *Necrolemur* and *Tetonius* as stem-haplorhines does not alter these interpretations (Fig. S6 in File S1). Taken together, these results reject a simple two-stepped grade-shift model of reduced olfactory sensitivity through primate phylogeny. If the phylogenetic model is a sufficient estimation, it provides an example of how a comparison of partitioned terminal taxa (Fig. 1) may obscure apomorphy and directionality in trait evolution.

## Supporting Information

File S1 **Supporting information. Table S1**, Olfactory bulb (OB) and total brain (TB) volumes. **Figure S1**, Crown-Primates subtree construction. **Figure S2**, Eulipotyphla subtree construction. **Figure S3**, Afroinsectivora subtree construction. **Figure S4**, Scandentia subtree construction. **Figure S5**, Final tree assembly. **Table S2**, Maximum likelihood values of different models and likelihood ratio comparisons. **Table S3**, Phylogenetic generalized least-squares model values and comparisons. **Table S4**, Reconstructed ancestral nodes. **Figure S6**, Ancestral reconstruction using an alternate tree.
(PDF)

File S2 **Supporting information.** DNA sequence accession numbers and concatenated alignments.
(ZIP)
