# Supplementary material for: Modeling Olfactory Bulb Evolution through Primate Phylogeny
Source: PLoS One. 2014 Nov 26;9(11):e113904. doi: 10.1371/journal.pone.0113904 (PMC4245229; doi:10.1371/journal.pone.0113904)

File S1 (Supporting Information)

Contents

Table S1	Olfactory bulb (OB) and total brain (TB) volumes.
Figure S1	Crown-Primates subtree construction.
Figure S2	Eulipotyphla subtree construction.
Figure S3	Afroinsectivora subtree construction.
Figure S4	Scandentia subtree construction.
Figure S5	Final tree assembly.
Table S2	Maximum likelihood values of different models and likelihood ratio comparisons.
Table S3	Phylogenetic generalized least-squares model values and comparisons.
Table S4	Reconstructed ancestral nodes.
Figure S6	Ancestral reconstruction using an alternate tree.

Table S1. Olfactory bulb (OB) and total brain (TB) volumes.

Species	Taxonomy			OB (mm^3)	TB (mm^3)	log_{10} OB	log_{10} TB
Galago demidoff	crown-Primates	Strepsirrhini	Lorisoidea	83.1	3203	1.919601	3.505557
Galago senegalensis	crown-Primates	Strepsirrhini	Lorisoidea	79.2	4512	1.898725	3.654369
Otolemur crassicaudatus	crown-Primates	Strepsirrhini	Lorisoidea	166.0	9668	2.220108	3.985337
Loris tardigradus	crown-Primates	Strepsirrhini	Lorisoidea	85.8	6269	1.933487	3.797198
Nycticebus coucang	crown-Primates	Strepsirrhini	Lorisoidea	159.0	11755	2.201397	4.070223
Perodicticus potto	crown-Primates	Strepsirrhini	Lorisoidea	310.0	13212	2.491362	4.120969
Daubentonia madagascariensis	crown-Primates	Strepsirrhini	Lemuroidea	685.0	42611	2.835691	4.629522
Varecia variegata	crown-Primates	Strepsirrhini	Lemuroidea	369.0	29713	2.567026	4.472947
Eulemur fulvus	crown-Primates	Strepsirrhini	Lemuroidea	207.0	22106	2.315970	4.344510
Lepilemur ruficaudatus	crown-Primates	Strepsirrhini	Lemuroidea	113.0	7175	2.053078	3.855822
Microcebus murinus	crown-Primates	Strepsirrhini	Lemuroidea	43.0	1680	1.633468	3.225309
Cheirogaleus major	crown-Primates	Strepsirrhini	Lemuroidea	158.0	6373	2.198657	3.804344
Cheirogaleus medius	crown-Primates	Strepsirrhini	Lemuroidea	102.0	2961	2.008600	3.471438
Indri indri	crown-Primates	Strepsirrhini	Lemuroidea	168.0	36285	2.225309	4.559727
Avahi laniger	crown-Primates	Strepsirrhini	Lemuroidea	89.2	9798	1.950365	3.991137
Avahi occidentalis	crown-Primates	Strepsirrhini	Lemuroidea	70.1	9124	1.845718	3.960185
Propithecus verreauxi	crown-Primates	Strepsirrhini	Lemuroidea	147.0	25194	2.167317	4.401297
*Adapis parisiensis**	crown-Primates	Strepsirrhini	stem-Strepsirrhini	266.6	8310	2.425860	3.919601
*Notharctus tenebrosus**	crown-Primates	Strepsirrhini	stem-Strepsirrhini	236.6	10430	2.374015	4.018284
Tarsius sp.	crown-Primates	Haplorhini	Tarsiiformes	18.8	3393	1.274158	3.530584
*Necrolemur antiquus**	crown-Primates	Haplorhini	stem-Tarsiiformes	51.3	2650	1.710117	3.423246
*Tetonius homunculus**	crown-Primates	Haplorhini	stem-Tarsiiformes	32.8	1500	1.515874	3.176091
*Parapithecus grangeri**	crown-Primates	Haplorhini	stem-Anthropoidea	75.0	11400	1.875061	4.056905
Pithecia monachus	crown-Primates	Haplorhini	Platyrrhini	34.8	32867	1.541579	4.516760
Callicebus moloch	crown-Primates	Haplorhini	Platyrrhini	19.2	17944	1.283301	4.253919
Alouatta sp.	crown-Primates	Haplorhini	Platyrrhini	41.4	49009	1.617000	4.690276
Lagothrix lagotricha	crown-Primates	Haplorhini	Platyrrhini	73.4	95503	1.865696	4.980017
Ateles geoffroyi	crown-Primates	Haplorhini	Platyrrhini	90.4	101034	1.956168	5.004468
Saimiri sciureus	crown-Primates	Haplorhini	Platyrrhini	26.8	22572	1.428135	4.353570
Cebus sp.	crown-Primates	Haplorhini	Platyrrhini	39.9	66939	1.600973	4.825679
Saguinus oedipus	crown-Primates	Haplorhini	Platyrrhini	19.1	9537	1.281033	3.979412
Saguinus midas	crown-Primates	Haplorhini	Platyrrhini	17.3	9569	1.238046	3.980867
Callimico goeldii	crown-Primates	Haplorhini	Platyrrhini	27.0	10510	1.431364	4.021603
Callithrix jacchus	crown-Primates	Haplorhini	Platyrrhini	22.8	7241	1.357935	3.859799
Callithrix pygmaea	crown-Primates	Haplorhini	Platyrrhini	12.3	4302	1.089905	3.633670
Aotus trivirgatus	crown-Primates	Haplorhini	Platyrrhini	56.0	16195	1.748188	4.209381
*Aegyptopithecus zeuxis**	crown-Primates	Haplorhini	stem-Catarrhini	102.0	14600	2.008600	4.164353
Pan troglodytes	crown-Primates	Haplorhini	Catarrhini	257.0	382103	2.409933	5.582180
Homo sapiens	crown-Primates	Haplorhini	Catarrhini	114.0	1251847	2.056905	6.097551
Gorilla gorilla	crown-Primates	Haplorhini	Catarrhini	316.0	470359	2.499687	5.672429
Hylobates lar	crown-Primates	Haplorhini	Catarrhini	43.9	97505	1.642465	4.989027
Macaca mulatta	crown-Primates	Haplorhini	Catarrhini	84.3	87896	1.925828	4.943969
Papio anubis	crown-Primates	Haplorhini	Catarrhini	287.0	190957	2.457882	5.280936
Lophocebus albigena	crown-Primates	Haplorhini	Catarrhini	121.0	97603	2.082785	4.989463
Erythrocebus patas	crown-Primates	Haplorhini	Catarrhini	51.8	103167	1.714330	5.013541
Cercopithecus mitis	crown-Primates	Haplorhini	Catarrhini	117.0	70564	2.068186	4.848583
Cercopithecus ascanius	crown-Primates	Haplorhini	Catarrhini	99.5	63505	1.997823	4.802808
Miopithecus talapoin	crown-Primates	Haplorhini	Catarrhini	27.8	37776	1.444045	4.577216
Nasalis larvatus	crown-Primates	Haplorhini	Catarrhini	30.3	92797	1.481443	4.967534
Pygathrix nemaeus	crown-Primates	Haplorhini	Catarrhini	10.7	72530	1.029384	4.860518
Piliocolobus badius	crown-Primates	Haplorhini	Catarrhini	51.3	73818	1.710117	4.868162
*Ignacius graybullianus**	stem-Primates	Paraomomyoidea		118.31	2141.288	2.073021	3.330675
*Microsyops annectens**	stem-Primates	Microsyopidae		300.554	5897.76	2.477923	3.770687
Cynocephalus sp.	Dermoptera	Cynocephalidae		130.5	5781.1	2.115611	3.762010
Tupaia glis	Scandentia	Tupaiidae		131.5	2999	2.118926	3.476976
Urogale everetti	Scandentia	Tupaiidae		184.0	3997	2.264818	3.601734
Tupaia minor	Scandentia	Tupaiidae		94.3	2430	1.974512	3.385606
Crocidura russula	Eulipotyphla	Soricidae	Crocidurinae	16.3	178	1.212188	2.250420
Crocidura olivieri	Eulipotyphla	Soricidae	Crocidurinae	37.6	408	1.575188	2.610660
Suncus murinus	Eulipotyphla	Soricidae	Crocidurinae	34.2	354	1.534026	2.549003
Neomys fodiens	Eulipotyphla	Soricidae	Soricinae	15.8	299	1.198657	2.475671
Sorex araneus	Eulipotyphla	Soricidae	Soricinae	15.7	188	1.195900	2.274158
Sorex minutus	Eulipotyphla	Soricidae	Soricinae	8.7	103	0.937016	2.012837
Hemiechinus auritus	Eulipotyphla	Erinaceidae		175.0	1710	2.243038	3.232996
Erinaceus europaeus	Eulipotyphla	Erinaceidae		332.0	3050	2.521138	3.484300
Atelerix algirus	Eulipotyphla	Erinaceidae		285.0	2859	2.454845	3.456214
Talpa europaea	Eulipotyphla	Talpidae		59.6	953	1.775246	2.979093
Galemys pyrenaicus	Eulipotyphla	Talpidae		39.0	1230	1.591065	3.089905
Desmana moschata	Eulipotyphla	Talpidae		141.0	3620	2.149219	3.558709
Solenodon paradoxus	Eulipotyphla	Solenodontidae		465.0	4282	2.667453	3.631647
Echinops telfairi	Afroinsectivora	Tenrecidae	Tenrecinae	71.2	566	1.852480	2.752816
Setifer setosus	Afroinsectivora	Tenrecidae	Tenrecinae	209.0	1404	2.320146	3.147367
Tenrec ecaudatus	Afroinsectivora	Tenrecidae	Tenrecinae	314.0	2315	2.496930	3.364551
Hemicentetes semispinosus	Afroinsectivora	Tenrecidae	Tenrecinae	93.6	757	1.971276	2.879096
Oryzorictes hova	Afroinsectivora	Tenrecidae	Oryzorictinae	46.9	538	1.671173	2.730782
Limnogale mergulus	Afroinsectivora	Tenrecidae	Oryzorictinae	42.9	1046	1.632457	3.019532
Microgale talazaci	Afroinsectivora	Tenrecidae	Oryzorictinae	74.3	741	1.870989	2.869818
Micropotamogale lamottei	Afroinsectivora	Tenrecidae	Potamogalinae	34.0	743	1.531479	2.870989
Potamogale velox	Afroinsectivora	Tenrecidae	Potamogalinae	86.0	3878	1.934498	3.588608
Chrysochloris asiatica	Afroinsectivora	Chrysochloridae		58.5	657	1.767156	2.817565
Chrysochloris stuhlmanni	Afroinsectivora	Chrysochloridae		60.6	693	1.782473	2.840733
Elephantulus fuscipes	Afroinsectivora	Macroscelididae		63.3	1233	1.801404	3.090963
Rhynchocyon cirnei	Afroinsectivora	Macroscelididae		426.0	5680	2.629410	3.754348

Data for extant taxa from Stephan et al. (1981) and Pirlot and Kamiya (1982). Data for fossil taxa (=*) from Bush et al. (1984), Gurche (1992), Silcox et al. (2009), Silcox et al. (2010) amd Simons et al. (2007).

Figure S1. Crown-Primates subtree construction.

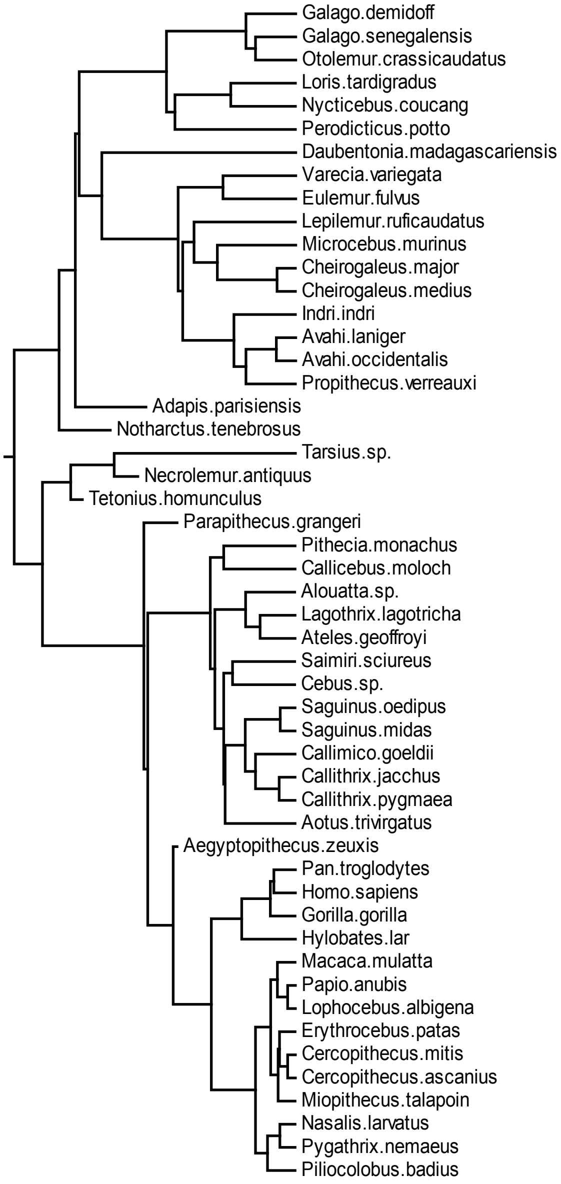

1. Chronogram (=ultrametric, time scaled) for extant primates followed Springer et al. (2012) [soft bounded, independent rates]. Taxa in the ordinal chronogram but not in the OB and TB dataset were pruned from the tree.

2. Six fossil crown-primates were grafted to the pruned chronogram of extant taxa. For fossil crown-primates (a) topological placements, (b) diversification dates, and (c) terminal dates followed Boyer and Seiffert (2013). Scale: 1.00 = 100 million years.

FOSSIL CROWN-PRIMATES:

1. *Adapis parisiensis*
Topological placement: sister to (crown-Strepsirrhini)
Diversification date: 0.559102
Terminal date: 0.38

2. *Notharctus tenebrosus*
Topological placement: sister to (*Adapis parisiensis*+crown-Strepsirrhini)
Diversification date: 0.6
Terminal date: 0.47

3. *Necrolemur antiquus*
Topological placement: sister to (*Tarsius*)
Diversification date: 0.46
Terminal date: 0.4

4. *Tetonius homunculus*
Topological placement: sister to (*Necrolemur antiquus*+*Tarsius*)
Diversification date: 0.57
Terminal date: 0.54

5. *Parapithecus grangeri*
Topological placement: sister to (crown-Anthropoidea)
Diversification date: 0.384136
Terminal date: 0.3

6. *Aegyptopithecus zeuxis*
Topological placement: sister to (crown-Catarrhini)
Diversification date: 0.31
Terminal date: 0.3

Newick format:

```
((((((Galago.demidoff:0.125971,(Galago.senegalensis:0.101979,Otolemur.crassicaudatus:0.10197999999999999):0.023992):
0.20165699999999998,((Loris.tardigradus:0.163744,Nycticebus.coucang:0.163745):0.141996,Perodicticus.potto:0.30574):0.021888):
0.221472,(Daubentonia.madagascariensis:0.491954,((Varecia.variegata:0.184421,Eulemur.fulvus:0.18442199999999997):0.113546,
((Lepilemur.ruficaudatus:0.25763800000000003,(Microcebus.murinus:0.198746,(Cheirogaleus.major:0.04682,Cheirogaleus.medius:
0.04682):0.151927):0.058891):0.028783,(Indri.indri:0.156182,((Avahi.laniger:0.048968,Avahi.occidentalis:0.048968):0.076767,
Propithecus.verreauxi:0.125736):0.030446):0.130239):0.011546):0.193988):0.057146):0.01,Adapis.parisiensis:0.179102):0.040898,
Notharctus.tenebrosus:0.13):0.113547,(((Tarsius.sp.:0.459999,Necrolemur.antiquus:0.06):0.11,Tetonius.homunculus:0.03):0.071795,
(Parapithecus.grangeri:0.084136,(((Pithecia.monachus:0.18110600000000004,Callicebus.moloch:0.18110700000000002):0.034283,
((Alouatta.sp.:0.12501400000000001,(Lagothrix.lagotricha:0.088702,Ateles.geoffroyi:0.088701):0.036312):0.07832,
((Saimiri.sciureus:0.15796700000000002,Cebus.sp.:0.157967):0.022722,(((Saguinus.oedipus:0.037793,Saguinus.midas:0.037793):
0.088336,(Callimico.goeldii:0.100564,(Callithrix.jacchus:0.040230999999999996,Callithrix.pygmaea:0.04023):0.060333):0.025564):
0.050936,Aotus.trivirgatus:0.177065):0.003625):0.022645):0.012055):0.158745,(Aegyptopithecus.zeuxis:0.01,((((Pan.troglodytes:
0.054377999999999996,Homo.sapiens:0.054378):0.008648,Gorilla.gorilla:0.063026):0.072995,Hylobates.lar:0.136021):0.076778,
(((Macaca.mulatta:0.045135,(Papio.anubis:0.019322,Lophocebus.albigena:0.019323):0.025813000000000003):0.016952,
((Erythrocebus.patas:0.039723,(Cercopithecus.mitis:0.019497,Cercopithecus.ascanius:0.019498):0.020225):0.003582,
Miopithecus.talapoin:0.043306):0.018781):0.038587,((Nasalis.larvatus:0.038777,Pygathrix.nemaeus:0.038777):0.031587000000000004,
Piliocolobus.badius:0.070363):0.03031):0.112126):0.097199):0.064136):0.01):0.257659):0.071752);
```

Figure S2. Eulipotyphla subtree construction.

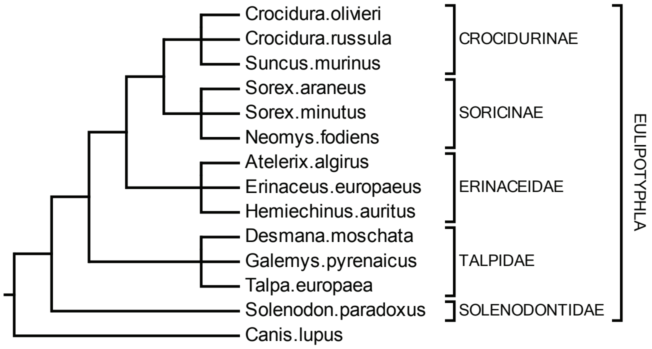

1. Constraint topology followed family (and subfamily) level DNA tree of Meredith et al. (2011). Taxa in the OB and TB volumetric dataset were binned in their respective taxonomic groups (assumes monophyly at these ranks).

Newick format:

```
(((((Crocidura.olivieri,Crocidura.russula,Suncus.murinus),
(Sorex.araneus,Sorex.minutus,Neomys.fodiens)),(Atelerix.algirus,
Erinaceus.europaeus,Hemiechinus.auritus)),(Desmana.moschata,
Galemys.pyrenaicus,Talpa.europaea)),Solenodon.paradoxus),
Canis.lupus);
```

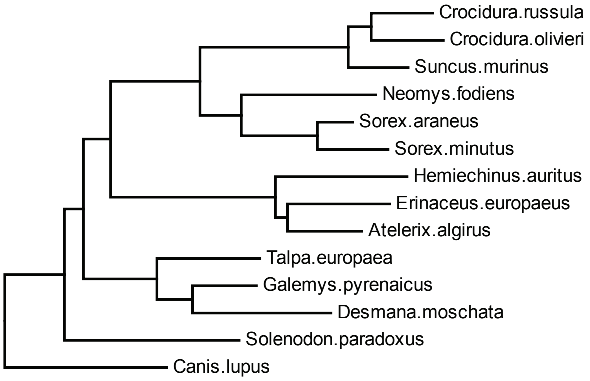

2. Phylogram (=genetic distance) produced by fitting a 13 gene (3 mitochondrial, 10 nuclear) concatenation to the constraint topology. Software: MEGA v5.10, Method: Maximum Likelihood, Model: GTR+G+I, Concatenation length: 11,777, Gene segments: 12S, COX1, CYTB, ADRAB2, APOB, BRCA1, CREM, EDG1, GHR, PLCB4, RAG1, RAG2, VWF.

Newick format:

```
((((((Crocidura.russula:0.04314379,Crocidura.olivieri:0.05055668):
0.01605615,Suncus.murinus:0.04205046):0.10400920,(Neomys.fodiens:
0.09444923,(Sorex.araneus:0.02462759,Sorex.minutus:0.04974728):
0.05332713):0.02911712):0.06282364,(Hemiechinus.auritus:0.09244562,
(Erinaceus.europaeus:0.07150381,Atelerix.algirus:0.05207989):
0.00868019):0.11580983):0.01925693,(Talpa.europaea:0.07233052,
(Galemys.pyrenaicus:0.04483551,Desmana.moschata:0.09721575):
0.02491691):0.05165280):0.01347531,Solenodon.paradoxus:0.12265715):
0.04188398,Canis.lupus:0.11359074);
```

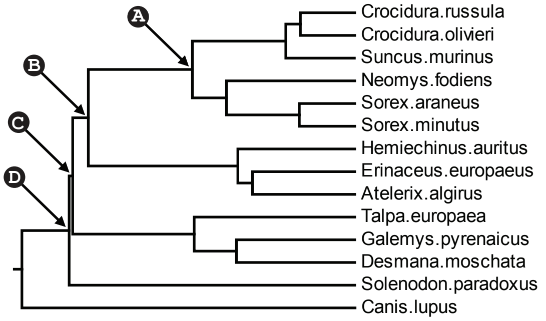

3. Chronogram (=ultrametric, time-scaled) produced by linearizing the phylogram with 4 fixed diversification dates. Dates followed Meredith et al. (2011) [DNA analysis, soft bounded, independent rates]. Software: r8s, Method: Penalized Likelihood & Newton algorithm, Scale: 1.00 = 100 million years. Outgroup was subsequently pruned from the subtree.

Newick format:

```
(((((((Crocidura.russula:0.145928,Crocidura.olivieri:0.145928):
0.038772,Suncus.murinus:0.184701):0.251499,(Neomys.fodiens:
0.344678,(Sorex.araneus:0.148752,Sorex.minutus:0.148752):0.195926):
0.091522):0.280500,(Hemiechinus.auritus:0.314198,
(Erinaceus.europaeus:0.275062,Atelerix.algirus:0.275062):0.039136):
0.402502):0.042300,(Talpa.europaea:0.432249,(Galemys.pyrenaicus:
0.318349,Desmana.moschata:0.318349):0.113900):0.326751):0.009900,
Solenodon.paradoxus:0.768900):0.127337,Canis.lupus:0.896237);
```

FIXED DIVERSIFICATION DATES:

A. 0.4362 Soricidae
(=Soricinae to Crocidurinae)

B. 0.7167 Erinaceidae to Soricidae

C. 0.7590 Talpidae to Erinaceidae+Soricidae

D. 0.7689 Eulipotyphla
(=Solenodontidae to Talpidae+Erinaceidae+Soricidae)

Figure S3. Afroinsectivora subtree construction.

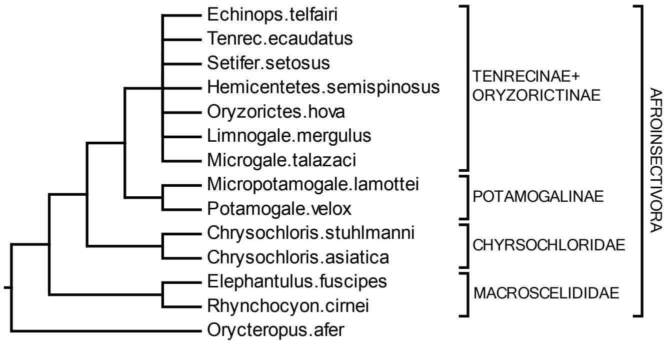

1. Constraint topology followed family (and subfamily) level DNA tree of Meredith et al. (2011). Taxa in the OB and TB volumetric dataset were binned in their respective taxonomic groups (assumes monophyly at these ranks).

Newick format:

```
(((((Echinops.telfairi,Tenrec.ecaudatus,Setifer.setosus,
Hemicentetes.semispinosus,Oryzorictes.hova,Limnogale.mergulus,
Microgale.talazaci),(Micropotamogale.lamottei,Potamogale.velox)),
(Chrysochloris.stuhlmanni,Chrysochloris.asiatica)),
(Elephantulus.fuscipes,Rhynchocyon.cirnei)),Orycteropus.afer);
```

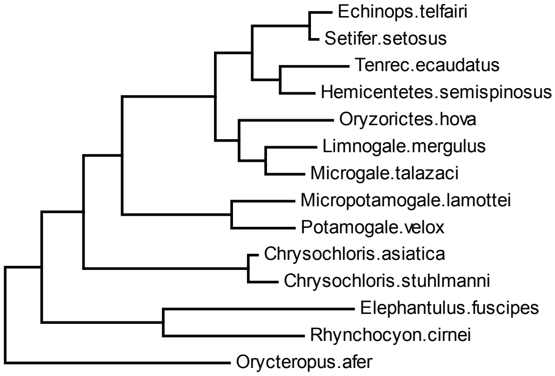

2. Phylogram (=genetic distance) produced by fitting an 11 gene (2 mitochondrial, 9 nuclear) concatenation to the constraint topology. Software: MEGA v5.10, Method: Maximum Likelihood, Model: GTR+G+I, Concatenation length: 11,708, Gene segments: 12S, ND2, ADRAB2, BDNF, BMI1, BRCA1, CREM, GHR, PLCB4, RAG1, VWF.

Newick format:

```
((((((Echinops.telfairi:0.01385078,Setifer.setosus:0.00557274):
0.03696563,(Tenrec.ecaudatus:0.04394974,Hemicentetes.semispinosus:
0.02198982):0.01798645):0.02385688,(Oryzorictes.hova:0.06042377,
(Limnogale.mergulus:0.03240558,Microgale.talazaci:0.02477554):
0.01717926):0.01535886):0.06020077,(Micropotamogale.lamottei:
0.04015078,Potamogale.velox:0.04042073):0.07060328):0.02485262,
(Chrysochloris.asiatica:0.00590999,Chrysochloris.stuhlmanni:
0.01883818):0.10624880):0.02701635,(Elephantulus.fuscipes:
0.12268446,Rhynchocyon.cirnei:0.09045019):0.07836542):0.05274231,
Orycteropus.afer:0.11569610);
```

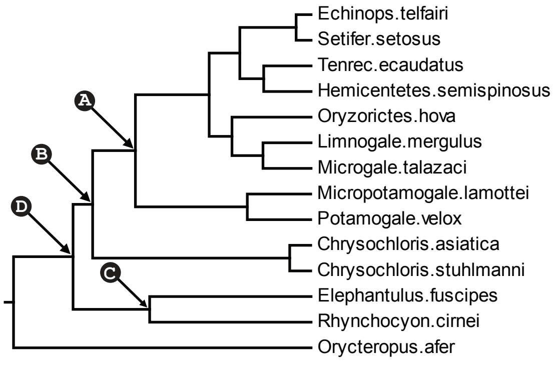

3. Chronogram (=ultrametric, time-scaled) produced by linearizing the phylogram with 4 fixed diversification dates. Dates followed Meredith et al. (2011) [DNA analysis, soft bounded, independent rates]. Software: r8s, Method: Penalized Likelihood & Newton algorithm, Scale: 1.00 = 100 million years. Outgroup was subsequently pruned from the subtree.

Newick format:

```
((((((Echinops.telfairi:0.048571,Setifer.setosus:0.048571):
0.182338,(Tenrec.ecaudatus:0.153568,Hemicentetes.semispinosus:
0.153568):0.077341):0.099501,(Oryzorictes.hova:0.256820,
(Limnogale.mergulus:0.155915,Microgale.talazaci:0.155915):
0.100905):0.073589):0.238891,(Micropotamogale.lamottei:0.206090,
Potamogale.velox:0.206090):0.363210):0.138200,
(Chrysochloris.asiatica:0.068318,Chrysochloris.stuhlmanni:
0.068318):0.639182):0.063800,(Elephantulus.fuscipes:0.523200,
Rhynchocyon.cirnei:0.523200):0.248100):0.193597,
Orycteropus.afer:0.964897);
```

FIXED DIVERSIFICATION DATES:

A. 0.5693 Tenrecidae
(=Potamogalinae to Tenrecinae+Oryzorictinae)

B. 0.7075 Afrosoricida
(=Chrysochloridae to Tenrecidae)

C. 0.5232 Macroscelididae
(=Rhynchocyon to Elephantulus)

D. 0.7713 Afroinsectivora
(=Macroscelididae to Afrosoricida)

Figure S4. Scandentia subtree construction.

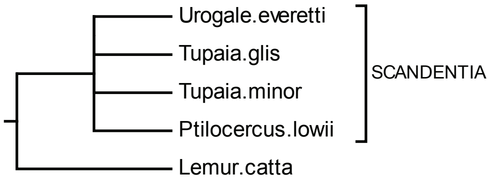

Newick format:

```
((Urogale.everetti,Tupaia.glis,Tupaia.minor,Ptilocercus.lowii),
Lemur.catta);
```

1. Constraint topology for the scandentian order (assumes monophyly at this rank). *U. everetti*, *T. glis* and *T. minor* are part of the OB and TB volumetric dataset. *P. lowii* is not part of the volumetric dataset but was included to provide an additional diversification date for the order.

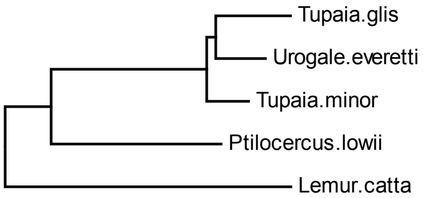

Newick format:

```
((((Tupaia.glis:0.03419161,Urogale.everetti:0.02278467):0.00409895,
Tupaia.minor:0.01909480):0.07120454,Ptilocercus.lowii:0.07777949):
0.02107726,Lemur.catta:0.13057236);
```

2. Phylogram (=genetic distance) produced by fitting a 14 gene (2 mitochondrial, 12 nuclear) concatenation to the constraint topology. Software: MEGA v5.10, Method: Maximum Likelihood, Model: GTR+G+I, Concatenation length: 9,850, Gene segments: 12S, 16S, ADORA3, ADRAB2, APP, ATP7A, BDNF, CREM, EDG1, PLCB4, PNOC, RAG2, TYR, VWF.

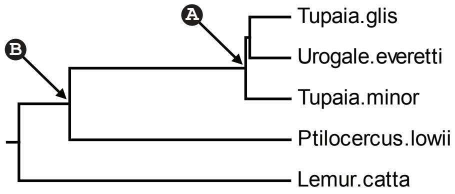

Newick format:

```
((((Tupaia.glis:0.102761,Urogale.everetti:0.102761):0.010739,
Tupaia.minor:0.113500):0.449000,Ptilocercus.lowii:0.562500):
0.129229,Lemur.catta:0.691729);
```

3. Chronogram (=ultrametric, time-scaled) produced by linearizing the phylogram with 2 fixed diversification dates. Date for *T. glis* to *T. minor* followed Springer et al. (2012) [soft bounded, independent rates]. Date for *Tupaia* to *P. lowii* followed Meredith et al. (2011) [DNA analysis, soft bounded, independent rates]. Software: r8s, Method: Penalized Likelihood & Newton algorithm, Scale: 1.00 = 100 million years. Outgroup and *P. lowii* were subsequently pruned from the subtree.

FIXED DIVERSIFICATION DATES:
A. 0.1135 *Tupaia glis* to *Tupaia minor*
B. 0.5625 *Tupaia* to *Ptilocercus lowii*

Figure S5. Final tree assembly.

The phylogeny used in this study (Fig. 2) was constructed by joining *Cynocephalus* (Dermoptera) and the four subtrees [crown-Primates (Fig. S1), Eulipotyphla (Fig. S2), Afroinsectivora (Fig. S3), Scandentia (Fig. S4)]. Topological relationships and diversification dates followed Meredith et al. (2011) [DNA analysis, soft bounded, independent rates]. Stem-Primates were then grafted to the tree following the topology of Bloch et al. (2007) and with branches terminating at specimen ages. Diversification for each stem-Primate branch was estimated at 1 Ma older than the base node of its sister-clade. Scale: 1.00 = 100 million years.

Topology:
((((crown-Primates,Dermoptera),Scandentia),Eulipotyphla),Afroinsectivora);

Diversification dates:
A. 0.8337 crown-Primates to Dermoptera
B. 0.8389 crown-Primates+Dermoptera to Scandentia
C. 0.9167 crown-Primates+Dermoptera+Scandenita to Eulipotyphla
D. 1.0099 crown-Primates+Dermoptera+Scandenita+Eulipotyphla to Afroinsectivora

STEM-PRIMATES

1. *Ignacius graybullianus*
Topological placement: sister to (crown-Primates)
Diversification date: 0.723547
Terminal date: 0.55

2. *Microsyops annectens*
Topological placement: sister to (crown-Primates+*Ignacius*)
Diversification date: 0.733547
Terminal date: 0.48

Final tree used in this study (Newick format):

```
(((((((((((((Galago.demidoff:0.125971,(Galago.senegalensis:0.101979,Otolemur.crassicaudatus:0.10197999999999999):0.023992):
0.20165699999999998,((Loris.tardigradus:0.163744,Nycticebus.coucang:0.163745):0.141996,Perodicticus.potto:0.30574):0.021888):
0.221472,(Daubentonia.madagascariensis:0.491954,((Varecia.variegata:0.184421,Eulemur.fulvus:0.18442199999999997):0.113546,
((Lepilemur.ruficaudatus:0.25763800000000003,(Microcebus.murinus:0.198746,(Cheirogaleus.major:0.04682,Cheirogaleus.medius:
0.04682):0.151927):0.058891):0.028783,(Indri.indri:0.156182,((Avahi.laniger:0.048968,Avahi.occidentalis:0.048968):0.076767,
Propithecus.verreauxi:0.125736):0.030446):0.130239):0.011546):0.193988):0.057146):0.01,Adapis.parisiensis:0.179102):0.040898,
Notharctus.tenebrosus:0.13):0.113547,(((Tarsius.sp.:0.459999,Necrolemur.antiquus:0.06):0.11,Tetonius.homunculus:0.03):0.071795,
(Parapithecus.grangeri:0.084136,(((Pithecia.monachus:0.18110600000000004,Callicebus.moloch:0.18110700000000002):0.034283,
((Alouatta.sp.:0.12501400000000001,(Lagothrix.lagotricha:0.088702,Ateles.geoffroyi:0.088701):0.036312):0.07832,
((Saimiri.sciureus:0.15796700000000002,Cebus.sp.:0.157967):0.022722,(((Saguinus.oedipus:0.037793,Saguinus.midas:0.037793):
0.088336,(Callimico.goeldii:0.100564,(Callithrix.jacchus:0.040230999999999996,Callithrix.pygmaea:0.04023):0.060333):0.025564):
0.050936,Aotus.trivirgatus:0.177065):0.003625):0.022645):0.012055):0.158745,(Aegyptopithecus.zeuxis:0.01,((((Pan.troglodytes:
0.054377999999999996,Homo.sapiens:0.054378):0.008648,Gorilla.gorilla:0.063026):0.072995,Hylobates.lar:0.136021):0.076778,
(((Macaca.mulatta:0.045135,(Papio.anubis:0.019322,Lophocebus.albigena:0.019323):0.025813000000000003):0.016952,
((Erythrocebus.patas:0.039723,(Cercopithecus.mitis:0.019497,Cercopithecus.ascanius:0.019498):0.020225):0.003582,
Miopithecus.talapoin:0.043306):0.018781):0.038587,((Nasalis.larvatus:0.038777,Pygathrix.nemaeus:0.038777):0.031587000000000004,
Piliocolobus.badius:0.070363):0.03031):0.112126):0.097199):0.064136):0.01):0.257659):0.071752):0.01,Ignacius.graybullianus:
0.173546):0.01,Microsyops.annectens:0.253546):0.100155,Cynocephalus.sp.:0.8337):0.0052,((Tupaia.glis:0.102761,Urogale.everetti:
0.102761):0.010739,Tupaia.minor:0.1135):0.7254):0.0778,((((((Crocidura.russula:0.145928,Crocidura.olivieri:0.145928):0.038772,
Suncus.murinus:0.184701):0.251499,(Neomys.fodiens:0.344678,(Sorex.araneus:0.148752,Sorex.minutus:0.148752):0.195926):0.091522):
0.2805,(Hemiechinus.auritus:0.314198,(Erinaceus.europaeus:0.275062,Atelerix.algirus:0.275062):0.039136):0.402502):0.0423,
(Talpa.europaea:0.432249,(Galemys.pyrenaicus:0.318349,Desmana.moschata:0.318349):0.1139):0.326751):0.0099,Solenodon.paradoxus:
0.7689):0.1478):0.0932,((((((Echinops.telfairi:0.048571,Setifer.setosus:0.048571):0.182338,(Tenrec.ecaudatus:0.153568,
Hemicentetes.semispinosus:0.153568):0.077341):0.099501,(Oryzorictes.hova:0.25682,(Limnogale.mergulus:0.155915,
Microgale.talazaci:0.155915):0.100905):0.073589):0.238891,(Micropotamogale.lamottei:0.20609,Potamogale.velox:0.20609):0.36321):
0.1382,(Chrysochloris.asiatica:0.068318,Chrysochloris.stuhlmanni:0.068318):0.639182):0.0638,(Elephantulus.fuscipes:0.5232,
Rhynchocyon.cirnei:0.5232):0.2481):0.2386):0.1,OG:1.1099);
```

Table S2. Maximum likelihood values of different models and likelihood ratio comparisons.

Olfactory Bulb

scaling parameters	Brownian Lh	Directional Lh
none	-36.500180	-36.296069
κ	-27.893537	-26.165441
δ	-33.799494	-33.535831
λ	-30.355702	-30.030860
κδ	-27.424841	-25.585347
κλ	-27.703511	-25.801022
δλ	-30.192024	-29.861930
κδλ	-27.353794	-25.460322

Brownian versions	LR	*P*
κ vs. none	**17.213286**	**0.000033**
κδ vs. κ	0.937392	0.332949
κλ vs. κ	0.380052	0.537575
κδλ vs. κ	1.079486	0.582898

Directional versions	LR	*P*
κ vs. none	**20.261256**	**0.000007**
κδ vs. κ	1.160188	0.281427
κλ vs. κ	0.728838	0.393260
κδλ vs. κ	1.410238	0.494050

InterModel	LR	*P*
Dir+κ vs. Bro+κ	3.456192	0.063015

Total Brain

scaling parameters	Brownian Lh	Directional Lh
none	-39.771246	-36.632103
κ	-33.903584	-33.028625
δ	-39.114350	-36.429743
λ	-39.489269	-36.228701
κδ	-33.706419	-32.611714
κλ	-33.903584	-33.028625
δλ	-39.070008	-36.187005
κδλ	-33.706419	-32.611714

Brownian versions	LR	*P*
κ vs. none	**11.735324**	**0.000613**
κδ vs. κ	0.394330	0.530032
κδλ vs. κ	0.394330	0.821055

Directional versions	LR	*P*
κ vs. none	**7.206956**	**0.007262**
κδ vs. κ	0.833822	0.361170
κδλ vs. κ	0.833822	0.659080

InterModel	LR	*P*
Dir+κ vs. Bro+κ	1.749918	0.185887

Lh = Maximum likelihood value
LR = Likelihood ratio value
Brownian, Bro = *BayesTraits* model A (constant variance random walk)
Directional, Dir = *BayesTraits* model B
Bold indicates signficant LR comparisons

Table S3. Phylogenetic generalized least-squares model values and comparisons.

Parameter Est	Model Lh	α (intercept)	β-2 (slope)	Var	R^2	s.e. α	s.e. β-2	Kappa (κ)	Delta (δ)	Lambda (λ)
Eulipotyphla										
none	8.960480	-1.141842	1.006294	0.034662	0.898954	0.322150	0.101723	fixed=1	fixed=1	fixed=1
*κ	10.977860	-1.059842	0.966355	0.345374	0.940405	0.217673	0.073348	ML=2.999986	fixed=1	fixed=1
δ	9.460576	-1.085986	0.992567	0.042488	0.900461	0.331391	0.099501	fixed=1	ML=0.448837	fixed=1
λ	8.960448	-1.141842	1.006294	0.034662	0.898954	0.322151	0.101723	fixed=1	fixed=1	ML=0.999995
Strepsirrhini										
*none	10.228534	-0.381396	0.650417	0.070715	0.769464	0.388656	0.091922	fixed=1	fixed=1	fixed=1
κ	10.360920	-0.315130	0.629770	0.136528	0.772559	0.371883	0.088228	ML=1.337308	fixed=1	fixed=1
δ	10.283485	-0.393929	0.652043	0.073142	0.766372	0.388664	0.092955	fixed=1	ML=1.323408	fixed=1
λ	10.228498	-0.381396	0.650417	0.070714	0.769463	0.388657	0.091923	fixed=1	fixed=1	ML=0.999995
Haplorhinni										
none	-7.273026	-0.961285	0.578220	0.727084	0.282785	0.883255	0.180594	fixed=1	fixed=1	fixed=1
κ	-2.298742	-0.454659	0.470195	0.030386	0.310912	0.561624	0.137281	ML=0.000000	fixed=1	fixed=1
δ	-4.666080	-0.821458	0.537017	0.665299	0.311995	0.729630	0.156395	fixed=1	ML=2.999998	fixed=1
*λ	-0.933177	-0.703902	0.509720	0.104737	0.597795	0.387711	0.081996	fixed=1	fixed=1	ML=0.000000

	Comparsion	LR	*P*
Eulipotyphla	κ vs. none	**4.034760**	**0.044572**
Strepsirrhini	κ vs. none	0.264772	0.606861
Haplorhini	λ vs. none	**12.679698**	**0.000370**

Values are from *BayesTraits* continuous PGLS regression.
Bold indicates significant likelihood ratio comparison.
Asterisk indicates model used for PGLS regression in Fig 1.

Table S4. Reconstructed ancestral nodes.

	Ancestral node	Olfactory bulb			Total brain			Ratio
		Mean	Lower 95% HPD	Upper 95% HPD	Mean	Lower 95% HPD	Upper 95% HPD	OB/TB
A	Afroinsectivora	2.0394	1.6693	2.4047	3.2544	2.8487	3.6717	0.6267
B	Boreoeutheria	2.1309	1.7851	2.4756	3.4011	3.0033	3.7958	0.6265
C	Eulipotyphla	2.1820	1.8134	2.5138	3.3488	2.9510	3.7441	0.6516
D	Euarchonta	2.1342	1.8084	2.4808	3.5139	3.1244	3.9042	0.6074
E	Scandentia	2.0995	1.8086	2.3894	3.4787	3.1301	3.8283	0.6035
F	crown-Primates	2.1212	1.7988	2.4052	3.6884	3.3287	4.0579	0.5751
G	Haplorhini	1.8642	1.5481	2.1892	3.6868	3.3021	4.0724	0.5056
H	pan-Anthropoidea	1.8557	1.5619	2.1388	4.0377	3.6798	4.3957	0.4596
I	Platyrrhini	1.6304	1.3341	1.9287	4.3682	4.0118	4.7338	0.3732
J	pan-Catarrhini	1.9283	1.6796	2.1721	4.3184	4.0012	4.6466	0.4465
K	Hominoidea	1.9350	1.6400	2.2299	5.0842	4.7205	5.4432	0.3806
L	Cercopithecoidea	1.7522	1.4594	2.0476	4.8318	4.4610	5.1915	0.3626
M	pan-Strepsirrhini	2.2762	1.9771	2.5725	3.8930	3.5359	4.2496	0.5847
N	Lemuroidea	2.4392	2.1011	2.7774	4.2295	3.8428	4.6222	0.5767
O	Lorisoidea	2.2345	1.9048	2.5711	3.9425	3.5583	4.3288	0.5668
	pan-Primates	2.1241	1.9026	2.5060	3.6332	3.2759	3.9882	0.5846
	crown-Strepsirrhini	2.3506	2.0379	2.6725	4.0382	3.6653	4.4169	0.5821
	pan-Tarsiiformes	1.6479	1.3788	1.9221	3.4293	3.0906	3.7798	0.4805
	Cercopithecinae	1.8201	1.5524	2.0853	4.8650	4.5169	5.1992	0.3741
	Colobinae	1.6059	1.3390	1.8720	4.8654	4.5346	5.1989	0.3301
	Placentalia	2.0936	1.7767	2.4078	3.3390	2.9764	3.7029	0.6270
	Root (alpha)	2.0935	1.7836	2.4030	3.3388	3.0032	3.6740	0.6270
	κ (scaling parameter)	0.3565	0.1467	0.5715	0.2684	0.0106	0.5193	

Values are $\log_{10}$ transformed volumes (mm^3).
HPD = highest posterior density.
BayesTraits settings are in methods.

Figure S6. Ancestral reconstruction using an alternate tree.

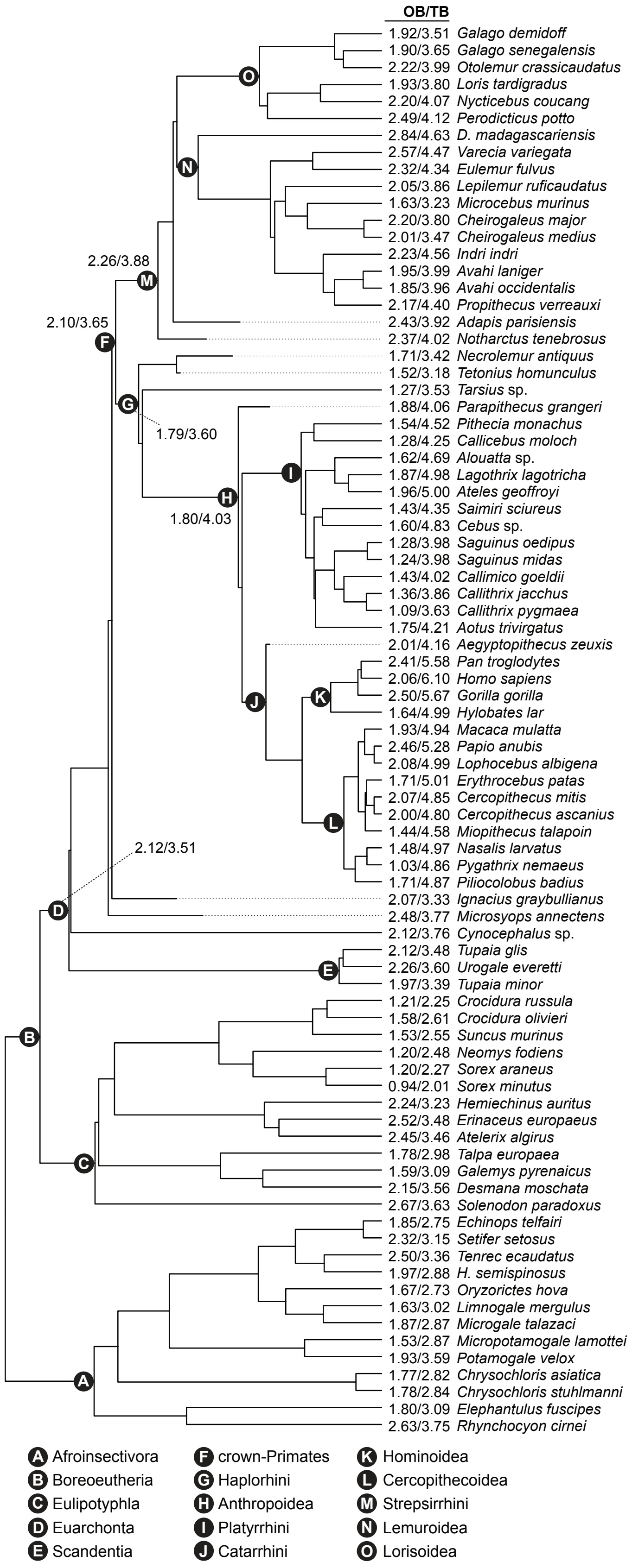

This alternate tree adapts the figure 2 tree by placing *Necrolemur* and *Tetonius* as stem-haplorhines. These two taxa where removed from the figure 2 tree and then re-grafted following the topology of Bajpai et al. (2008). For both taxa, branch termination dates were retained. Following Boyer and Seiffert (2013), ghost lineages were minimized by estimating diversification of (1) *Necrolemur*+*Tetonius* from crown-haplorhines at 1 Ma older than the crown-haplorhine node and (2) *Necrolemur* from *Tetonius* at 1 Ma older than the *Tetonius* terminal. Estimation of values at ancestral nodes using the alternate tree followed the same procedure described for the figure 2 tree (see methods).

Nodal reconstructions using different trees.

	Figure 2 tree		Alternate tree	
	OB	TB	OB	TB
D Euarchonta	2.13	3.51	2.12	3.51
F crown-Primates	2.12	3.69	2.10	3.65
G Haplorhini	1.86	3.69	1.79	3.60
H Anthropoidea	1.86	4.04	1.80	4.03
M Strepsirrhini	2.28	3.89	2.26	3.88

Paths-plots of OB evolution.
(A) OB change relative to TB volume; (B) absolute OB change. Vertical axes depict clade nesting and are dimensionless. Solid-outline circles represent nodal reconstructions using the figure 2 tree. Dashed-outline circles represent nodal reconstructions using the alternate tree.

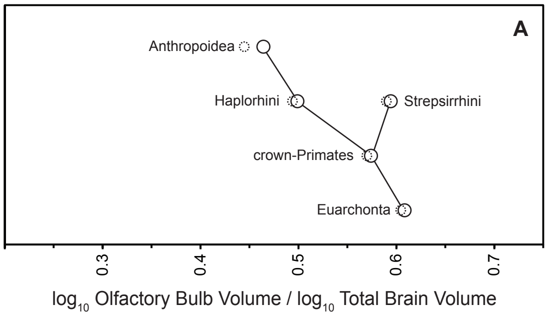

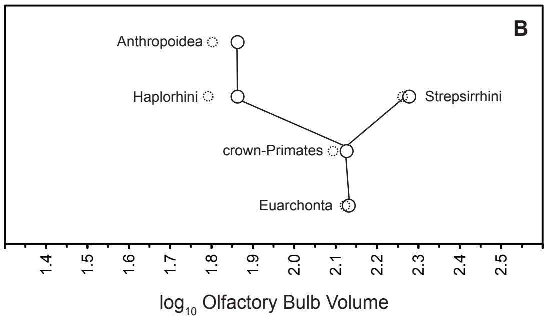

Supplement: File S1 — Supporting information. Table S1, Olfactory bulb (OB) and total brain (TB) volumes. Figure S1, Crown-Primates subtree construction. Figure S2, Eulipotyphla subtree construction. Figure S3, Afroinsectivora subtree construction. Figure S4, Scandentia subtree construction. Figure S5, Final tree assembly. Table S2, Maximum likelihood values of different models and likelihood ratio comparisons. Table S3, Phylogenetic generalized least-squares model values and comparisons. Table S4, Reconstructed ancestral nodes. Figure S6, Ancestral reconstruction using an alternate tree. (PDF) [file pone.0113904.s001.pdf]
